# Epidemiology and lifestyle survey of non-alcoholic fatty liver disease in school-age children and adolescents in Shenyang, Liaoning

**DOI:** 10.1186/s12887-022-03351-w

**Published:** 2022-05-17

**Authors:** Guan Lin, Zhang Xinhe, Tian Haoyu, Jin Xing, Li Dan, Wang Ningning, Sun Jing, Wang Xue, Zeng Zilu, Li Yiling

**Affiliations:** 1 Gastroenterology Department, The First Affiliated Hospital of China Medical University, No.155 North Nanjing Street, Heping District, Shenyang, 110001 Liaoning China; 2 3Rd Clinical Department, China Medical University, No.77 Puhe Road, Shenyang North New Area, Shenyang, 110122 Liaoning China

**Keywords:** NAFLD, Children, Adolescent, Epidemiology, Questionnaire

## Abstract

**Background:**

Non-alcoholic fatty liver disease (NAFLD) is diagnosed increasingly in children and adolescents. We aimed to investigate the prevalence and related influencing factors of NAFLD in school-aged children and adolescents in Shenyang, Liaoning Province. Furthermore, we analyzed the relationship between lifestyle and fatty liver.

**Methods:**

We conducted aprospective cohort study of 1309 school-aged children and adolescents between the ages of 7 and 18 years who underwent physical examination from November to December 2019. In addition, they were collected age, gender, learning stage, height, weight, BMI, waist circumference, hip circumference, and waist-hip ratio. Finally, a portion of the population was selected to complete a questionnaire survey to explore the impact of lifestyle habits on fatty liver disease.

**Results:**

NAFLD was present in 23.83% of subjects. The prevalence of children and adolescents was 22.73% and 24.43%, respectively. Fatty liver prevalence differs significantly by gender and learning stages. The highest rate of fatty liver was seen in obese children (71.68%). Moreover, exercise, diet, and parental factors can affect children with fatty liver.

**Conclusions:**

NAFLD is very prevalent in children and adolescents in Shenyang city. Due to the close relationship between NAFLD and obesity, lifestyle plays a major role in the occurrence of NAFLD.

**Trial registration:**

The First Affiliated Hospital of China Medical University, [2020] 2020–258-2. Registered 6 June 2020—Retrospectively registered.

**Supplementary Information:**

The online version contains supplementary material available at 10.1186/s12887-022-03351-w.

## Background

Nonalcoholic fatty liver disease (NAFLD) is the most common chronic liver disease in children and adolescents [1]; it refers to the abnormal accumulation of fat in the liver caused by reasons other than alcohol. The main risk factors include obesity, hyperlipidemia, hypertension, and insulin resistance [2]. The common clinical manifestations include irritability, fatigue, muscle soreness, cramps and headache [3]. However, most children and adolescents with NAFLD showed no symptoms. If nonalcoholic fatty liver is not controlled, it will further develop into nonalcoholic steatohepatitis (NASH), liver fibrosis, cirrhosis, and even liver cancer [4]. Furthermore, NAFLD is related not only to intrahepatic diseases but also to many extrahepatic diseases. One report showed that NAFLD could increase the risk of type 2 diabetes and atherosclerosis in children and adolescents and, in some cases, could even change the structure of the left ventricle of the heart and systolic and diastolic function [5]. It was currently found that NAFLD might also be correlated with changes in intestinal flora, obstructive sleep, apnea, and polycystic ovary syndrome [6]. Liver biopsy is the gold standard for the diagnosis of NAFLD [7]. However, it is rarely used in clinical practice. At present, the most universal test is liver ultrasound. For treatment, weight loss is the most effective method for the treatment of nonalcoholic fatty liver disease in children and adolescents. Although drug treatment options are relatively limited, they show great promise for development. Moreover, vitamin E has now been proven to improve the histology of NASH in children and adolescents [8].

School age is a critical period of growth and development. Therefore, the physical state at this stage is particularly important and is the basis of a normal learning life [9]. However, due to the improvement of modern socioeconomic conditions and changes in dietary patterns, the prevalence of NAFLD in this part of the population is increasing [10]. This phenomenon seriously affects normal life and learning, which has become an urgent problem. There are few epidemiological studies on NAFLD in children. In a meta-analysis [11], the global prevalence of NAFLD in children is 7.6%. The prevalence of NAFLD is different in children of different genders and ages, and even the prevalence of obese children is as high as 34.2%. At the same time, the prevalence of NAFLD in children in different regions is still different. The prevalence rate in Asia is 5.9%. Among obese children, the prevalence rate of NAFLD in Asia is higher than that in Europe and North America [12]. There is no report on the NAFLD data of school-aged children and adolescents in Shenyang, Liaoning Province. Therefore, this article reports the prevalence of NAFLD for the first time. By analyzing the associated risk factors and lifestyle, we explored the factors affecting the occurrence of NAFLD. This study can provide new ideas for the diagnosis and treatment of NAFLD in school-aged children and adolescents.

## Methods

### Study data

This study was conducted in Shenyang, Liaoning province from November to December 2019 as a prospective cohort study. By stratified random sampling, we numbered 10 districts and 2 districts were randomly selected. Then, each district randomly selected one elementary school, one middle school, and one high school. Finally, two classes were randomly selected at each grade level of the selected school. All students aged between 7 to 18 years old and had been living in Shenyang for a period of 5 years or more. At last, 1309 students were conducted physical examination. The following students were excluded: (1) Those who were allergic to the couplant; (2) Those who did not cooperate examination; (3) Thoses who had incomplete datawere. Thus, the data analysis was only performed on the remaining 1301 students, accounting for 1.58‰ of total student population in Shenyang. The study was approved by the First Affiliated Hospital of China Medical University ([2020]258).

### Anthropometric measurement

We used a homemade epidemiological questionnaire to record school attendance, grade, education, gender, and age. Height, weight, waist circumference, hip circumference were measured in the morning on an empty stomach. Body mass index (BMI) was calculated by using height and weight (BMI = weight (kg)/square of height (m^2^)). Waist-to-hip ratio (WHR) was calculated by using waist circumference and hip circumference (WHR = waist circumference (cm)/hip circumference (cm)).

### Fatty liver examination

Fatty liver examination was performed by two well-trained doctors, using transient elastography (Fibroscan). Fibroscan is newly noninvasive device for liver examination. The detection is fast and accurate, especially in the detection of fatty liver. It can not only provide quantitative results of fat but also display information on liver stiffness. Refering to the user manual, the subjects were divided into non-NAFLD (CAP value < 238 dB /m), mild fatty liver (238 dB /m ≤ CAP value < 259 dB /m), moderate fatty liver (259 dB /m ≤ CAP value < 292 dB /m) and severe fatty liver (CAP value ≥ 292 dB /m) according to the controlled attenuation parameter (CAP) value [13].

### Questionnaire

We randomly selected three classes from elementary school, middle school and high school among the enrolled students; the parients completed the questionnaire created by the First Affiliated Hospital of China Medical University on the lifestyles of children and adolescents. The questionnaire included basic information and information on the respondents’ birth, movement, entertainment, diet, learning condition, and awareness of parents. The valid questionnaires were divided into a fatty liver group and a non-fatty liver group according to the test results. The relationship between fatty liver disease and living habits was compared in children (7–12 years) and adolescents (13–18 years).

### Statistics analysis

The students included in the study were grouped according to gender, learning stage, overweight, obesity and NAFLD. The data of the study were analyzed by SPSS 23 statistical software. Measurement data were expressed as the mean ± standard deviation, and the t-test was performed. The count data were expressed as percentages (%) or proportions, and Chi square test was performed. If *p* < 0.05, the difference was statistically significant. Univariate and Multivariate Statistical Analysis were used for the risk factors of fatty liver. In univariate analysis, factors with *p* < 0.05 were included in multivariate analysis. If *p* < 0.05 in multivariate analysis, the OR value is statistically significant. OR > 1 indicates that this factor is a risk factor for fatty liver. OR < 1 indicates that this factor is a protective factor for fatty liver.

## Results

### Basic information

The average of age among the participants was 13.31 ± 2.98 years old. The number of patients with fatty liver was 310, and the total prevalence was 23.83% (95% CI: 26.1% -21.5%); 22.73% (95% CI: 18.9%-26.6%) of the children and 24.43% (95% CI: 21.5%- 27.3%) of the adolescents were diagnosed with NAFLD. The age, height, weight, BMI, waist circumference, hip circumference, WHR, CAP, liver stiffness, and prevalence of mild to severe fatty liver are detailed in Table 1.

**Table 1:** Analysis of basic data of school-age children, adolescents and the total population

Research factors	Total	Children	Adolescents
n	1301	462	839
Prevalence(n/%)	310/23.83%	105/22.73%	205/24.43%
Prevalence of mild fatty liver(n/%)	139/10.68%	66/14.29%	73/8.7%
Prevalence of moderate fatty liver(n/%)	100/7.69%	29/6.28%	71/8.46%
Prevalence of severe fatty liver(n/%)	71/5.46%	10/2.16%	61/7.27%
Age(years)	13.31 ± 2.98	9.92 ± 1.78	15.18 ± 1.49
Height(cm)	158.29 ± 15.66	143.23 ± 14.23	166.59 ± 8.67
Weight(kg)	55.12 ± 19.81	40.73 ± 16.44	63.04 ± 16.82
Waist(cm)	70.82 ± 13.07	66.51 ± 13.14	73.19 ± 12.41
Hip(cm)	90.05 ± 12.63	81.71 ± 11.72	94.63 ± 10.60
CAP(dB/m)	220.63 ± 39.41	215.15 ± 35.37	223.18 ± 41.22
Liver stiffness(kPa)	6.03 ± 2.28	5.57 ± 2.34	6.29 ± 2.20
BMI(kg/m^2^)	21.35 ± 5.23	19.16 ± 4.86	22.56 ± 5.03
WHR	0.79 ± 0.08	0.81 ± 0.08	0.77 ± 0.07
Overweight(n/%)	238/18.29%	80/17.32%	158/18.83%
Obesity(n/%)	279/21.45%	105/22.73%	174/20.74%

*BMI* body mass index, *WHR* waist-to-hip ratio

### Different learning stages, gender and NAFLD

In this study, there were 395 elementary school students, 427 middle school students, and 479 high school students, among whom there were 91, 107, and 112 fatty liver patients, respectively; the prevalence rates were 23.04% (95% CI: 0.189–0.272), 25.06% (95% CI: 0.209–0.292) and 23.38% (95% CI: 0.196–0.272), respectively. The results showed that there was no significant difference in the prevalence of the three stages (*p* > 0.05). Most of the elementary school students with fatty liver had mild fatty liver, accounting for 15.19%. However, the proportion of junior high school students with moderate fatty liver was the highest. Nonetheless, there was no significant difference in the prevalence of moderate fatty liver among the three grades (*p* = 0.023). The prevalence of severe fatty liver increased significantly with higher education (*p* < 0.001). The univariate analysis showed that there was no difference between junior high school students and senior high school students in CAP or liver stiffness. However, both groups were significantly higher on these measures than were elementary school students (*p* < 0.001). In addition, the other factors increased significantly with the improvement of academic qualifications (*p* < 0.001) (Table 2).

**Table 2:** Comparison of prevalence and related factors among different grade and sex

	*Grade level*				*Sex*		
	Primary school students	Middle school students	High school students	*P* value	Boys	Girls	*P* value
n	395	427	479		345/26.52%	956/73.48%	
Prevalence(n/%)	91/23.04%	107/25.06%	112/23.38%	> 0.05	182/29.12%	128/18.93%	< 0.001
Prevalence of mild fatty liver(n/%)	60/15.19%	38/8.9%	41/8.56%	< 0.001	77/12.32%	62/9.17%	0.066
Prevalence of moderate fatty liver(n/%)	23/5.82%	45/10.54%	32/6.68%	0.023	54/8.64%	46/6.8%	0.214
Prevalence of severe fatty liver(n/%)	8/2.03%	24/5.62%	39/8.14%	< 0.001	51/8.16%	20/2.96%	< 0.001
Age(years)	9.57 ± 1.68	13.50 ± 0.97	16.23 ± 0.91	< 0.001	13.29 ± 2.84	13.33 ± 3.11	0.823
Height(cm)	140.51 ± 13.37	163.89 ± 8.67	167.97 ± 8.36	< 0.001	162.73 ± 16.42	154.19 ± 13.73	< 0.001
Weight(kg)	38.26 ± 15.74	59.31 ± 16.55	65.27 ± 16.27	< 0.001	60.15 ± 21.82	50.46 ± 16.45	< 0.001
BMI(kg/m^2^)	18.73 ± 4.83	21.89 ± 4.96	23.03 ± 4.94	< 0.001	22.05 ± 5.64	20.70 ± 4.73	< 0.001
Waist(cm)	65.95 ± 13.13	71.47 ± 12.61	74.27 ± 12.19	< 0.001	74.60 ± 13.98	67.33 ± 11.08	< 0.001
Hip(cm)	80.23 ± 11.55	91.50 ± 9.87	96.84 ± 10.43	< 0.001	92.01 ± 12.76	88.23 ± 12.23	< 0.001
WHR	0.82 ± 0.08	0.78 ± 0.08	0.76 ± 0.07	< 0.001	0.81 ± 0.08	0.76 ± 0.07	< 0.001
CAP(dB/m)	214.58 ± 35.34	222.30 ± 40.52	223.31 ± 41.14	0.002	226.01 ± 41.81	215.07 ± 36.31	< 0.001
Liver stiffness(kPa)	5.54 ± 2.48	6.13 ± 2.56	6.36 ± 1.70	< 0.001	6.22 ± 2.43	5.87 ± 2.12	0.006

The 1301 study subjects included 625 boys and 676 girls. There were 182 boys with fatty liver, and the prevalence was 29.12% (95% CI: 0.255–0.327); there were 128 girls with fatty liver, and the prevalence was 18.93% (95% CI: 0.160–0.219). The prevalence of boys was significantly higher than that of girls (*p* < 0.001), which was mainly reflected in the prevalence of severe fatty liver. The results of univariate analysis showed that there was no statistically significant difference in age between the two groups. However, the values of height, weight, BMI, waist circumference, hip circumference, WHR, CAP (*p* < 0.001), and liver stiffness (*p* < 0.01) indicators were significantly higher in boys (Table 2). The overall prevalence of fatty liver increases with age; for instance, the prevalence of fatty liver in students younger than 8 years old is 2.38%, and the prevalence in students older than 17 years old is 24.76%. The prevalence was also different for boys and girls of different ages. After stratification by sex and age, only the 13–14 age group showed that the prevalence among boys was significantly higher than that among girls (*p* < 0.001). In addition, although the prevalence among boys was higher than that among girls at other ages, there was no statistical significance. Similarly, after stratification by gender and education, the prevalence of boys in elementary school and middle schools was significantly higher than that of girls (*p* < 0.05). However, there was no difference between the two groups in high school (Table 3).

**Table 3:** Comparison of prevalence of NAFLD under the delamination of gender, grade level and age

Variable	Total(n)	(%)	Boys(n)	(%)	Girls(n)	(%)	*P* value
Age(years)							
7–8	22/84	2.38%	11/37	29.73%	11/47	23.40%	0.513
9–10	38/195	19.49%	20/84	23.81%	18/111	16.26%	0.185
11–12	45/183	24.59%	27/92	29.35%	18/91	19.78%	0.133
13–14	64/279	22.94%	49/159	30.82%	15/120	12.50%	< 0.001
15–16	90/354	25.42%	52/178	29.21%	38/176	2.59%	0.1
17–18	51/206	24.76%	22/75	29.33%	29/131	22.14%	0.25
Grade level							
Elementary school students	91/395	23.04%	56/194	28.87%	35/201	17.41%	0.007
Middle school students	107/427	25.06%	70/218	32.11%	37/209	17.70%	0.001
High school students	112/479	23.38%	56/213	26.29%	56/266	21.05%	0.178

*P* value: comparison between boys and girls

### Analysis of risk factors for NAFLD

According to the Fibrotouch results, 1301 study subjects were divided into NAFLD group (310 cases) and non-NAFLD group (991 cases). After univariate statistical analysis, the results showed that in children, adolescents or all students, height, weight, waist and hip circumference, BMI, and WHR in NAFLD group were significantly higher than those in the non-NAFLD group (*p* < 0.001). The number of obesity and the proportion of boys in NAFLD group were more than those in the non-NAFLD group (*p* < 0.001). However, there was no statistically significant difference between the two groups in terms of age and the number of overweight. Furthermore, in the multivariable logistic regression analysis, it was also shown that boys, height, weight, BMI, waist and hip circumference, obesity are risk factors for NAFLD in children, adolescents or all students (Tables 4 and 5).

**Table 4:** Univariate statistical analysis of NAFLD risk factors

	Chidren			Adolescents			Total		
	non-NAFLD NAFLD *P* value			non-NAFLD NAFLD *P* value			non-NAFLD NAFLD *P* value		
n	357	105		634	205		991	310	
Gender (boys/girls)	154/203	59/46	0.018	289/345	123/82	< 0.001	443/548	182/128	< 0.001
Age(years)	9.90 ± 1.76	9.97 ± 1.83	0.735	15.17 ± 1.48	15.22 ± 1.52	0.681	13.27 ± 2.99	13.44 ± 2.97	0.386
Height(cm)	141.93 ± 13.43	147.64 ± 15.99	0.001	165.77 ± 8.65	169.12 ± 8.27	< 0.001	157.18 ± 15.61	161.84 ± 15.32	< 0.001
Weight(kg)	36.90 ± 11.79	53.72 ± 22.40	< 0.001	56.98 ± 11.26	81.77 ± 17.38	< 0.001	49.75 ± 14.97	72.27 ± 23.35	< 0.001
BMI(kg/m^2^)	17.89 ± 3.33	23.45 ± 6.52	< 0.001	20.65 ± 3.31	28.45 ± 4.49	< 0.001	19.66 ± 3.57	26.76 ± 5.97	< 0.001
Waist(cm)	63.57 ± 10.44	76.51 ± 16.17	< 0.001	68.75 ± 8.12	86.93 ± 13.32	< 0.001	66.89 ± 9.35	83.40 ± 15.15	< 0.001
Hip(cm)	79.36 ± 9.43	89.70 ± 14.85	< 0.001	91.38 ± 8.82	104.71 ± 9.24	< 0.001	87.05 ± 10.73	99.62 ± 13.46	< 0.001
WHR	0.80 ± 0.08	0.85 ± 0.07	< 0.001	0.75 ± 0.06	0.83 ± 0.08	< 0.001	0.77 ± 0.07	0.83 ± 0.08	< 0.001
Overweight(n/%)	65/18.21%	15/14.29%	0.351	115/18.14%	43/20.98%	0.366	180/18.16%	58/18.71%	0.828
Obesity(n/%)	44/12.32%	61/58.10%	< 0.001	35/5.52%	139/67.80%	< 0.001	79/7.97%	200/64.52%	< 0.001

*BMI* body mass index, *WHR* waist-to-hip ratio

*P* value: comparison between non-fatty liver group and fatty liver group

**Table 5:** Multivariable statistical analysis of NAFLD risk factors

	Chidren			Adolescents			Total		
	*P* value	OR	95%CI	*P* value	OR	95%CI	*P* value	OR	95%CI
Gender	0.019	1.691	1.090–2.622	< 0.001	1.791	1.300–2.466	< 0.001	1.759	1.358–2.2.78
Height	< 0.001	1.03	1.013–1.047	< 0.001	1.046	1.027–1.066	< 0.001	1.021	1.012–1.030
Weight	< 0.001	1.066	1.049–1.082	< 0.001	1.137	1.116–1.158	< 0.001	1.073	1.063–1.083
BMI	< 0.001	1.290	1.219–1.366	< 0.001	1.608	1.503–1.721	< 0.001	1.399	1.344–1.456
Waist	< 0.001	1.088	1.066–1.111	< 0.001	1.179	1.152–1.208	< 0.001	1.132	1.115–1.150
Hip	< 0.001	1.085	1.062–1.109	< 0.001	1.184	1.154–1.215	< 0.001	1.107	1.091–1.123
WHR	0.073	-	-	< 0.001	1.193	1.172–1.203	< 0.001	1.197	1.021–1.281
Obesity	< 0.001	1.048	1.034–1.066	< 0.001	1.048	1.029–1.078	< 0.001	1.068	1.048–1.097

## Questionnaire

The questionnaire was valid for 117 of the 123 students. Supplementary table 1 and 2 present the statistical and correlation analyses of various factors in the fatty liver group and non-fatty liver group. The basic information of children is consistent with that shown in the epidemiological survey. More fathers graduated from high school and below in the NAFLD group (*p* < 0.05). Regarding exercise habits, the proportion of students in the NAFLD group who did not exercise or who had poor grade was higher. In terms of eating habits, the students in the NAFLD group ate breakfast less frequently than those in the non-NAFLD group (*p* < 0.05). However, the frequency of fast food and snacks was higher (*p* < 0.05). Furthermore, the ratio of eating carefully and slowly was higher in the non-NAFLD group. Parents of adolescents who had NAFLD had higher BMI. Similar to children, more students ate breakfast every day in non-NAFLD group (*p* < 0.05). In particular, parents of adolescents with NAFLD received less information on nutrition and health from the community. Finally, no statistically significant differences were found between the two groups in basic conditions of birth, recreation and learning (*p* > 0.05).

## Discussion

Many studies have investigated the prevalence of NAFLD. Jeffery conducted a retrospective survey of 742 children aged 2–19 years old from 1993 to 2003. The study found that the average prevalence of fatty liver was 9.6% [14]. A 15-year meta-analysis showed that the average prevalence of fatty liver in children and adolescents aged 1–19 years was 7.6% [7]. Different races have different prevalence rates; the prevalence among children and adolescents in Asia was 10.2% [9]; the prevalence rate in Europe was 2.5% [15]; and the prevalence rate among Hispanics was 11.8%, which is the highest prevalence rate. Ajay Jain also demonstrated this point in a recent dissertation [16], and different countries also have different prevalence rates; for example, in Haryana, India, 22.4% of children aged 5–10 years had fatty liver [17], and the average prevalence of fatty liver in Chinese children is 9.03% [18]. In our study, the prevalence among school-aged children and adolescents in Shenyang was 23.83%, which is higher than the national average. This phenomenon is likely to be related to diet and lifestyle. Many children and adolescents in this area prefer sweets and meaty diets. In addition, the weather in the area is generally cold, which causes most children and adolescents to spend more time indoors, and indoor exercise is limited.

This study found that 64.93% of overweight children and adolescents in Shenyang had fatty liver, which is significantly higher than that of non-overweight people. A study in Beijing showed that 174 of 387 obese children and adolescents had nonalcohol fatty liver, with a prevalence of 45.0% [19]. Another document in China also reported that the prevalence among 308 obese children aged 9–14 was 65.9% [20]. In the univariate analysis, we also found that the prevalence of fatty liver was closely related to overweight, obesity, and abdominal obesity. The results of these studies indicated that overweight and obese individuals had a higher probability of developing fatty liver. Furthermore, it was reported that fatty acids were mainly catabolized in the liver [21]. Obese children and adolescents will have increased levels of fatty acids entering the liver, which will exceed the liver's metabolism. Thus, it can cause the deposition of a large number of fat droplets in the liver, which make liver dysfunction and promote the formation of fatty liver. In addition, free fatty acids will also increase the release of inflammatory mediators and insulin resistance [22]. These factors increase the production of intrahepatic fat [23]. Among obese people, central obesity (abdominal obesity) mainly reflects the amount of visceral fat, which is more dangerous than peripheral obesity is. In our research, non-overweight children and adolescents still had a 9% prevalence of fatty liver. Fatty liver in non-overweight people increases the risk of cardiac metabolic disease, causing more serious consequences. On the one hand, it may be related to genetic factors. Many genetic variations have been found to be associated with fatty liver. PNPLA3 gene polymorphisms can increase liver fat content, increase the risk of NAFLD and even be related to the severity of NAFLD [24]. TM6SF2, MBOAT7, or GCKR can all affect the occurrence and development of NAFLD in children and adolescents [25]. On the other hand, fatty liver may be connected with irregular lifestyles; excessive intake of fructose will promote the formation of NAFLD, although this will not cause obesity in some people [26]. Adult studies have found that elevated serum uric acid could increase the risk of lean NAFLD [27]. However, further exploration is needed to determine whether this conclusion is equally applicable to children and adolescents.

We found that boys showed a higher prevalence of NAFLD than girls did, and this conclusion is the same as that of most studies. According to previous studies, estrogen plays a major role in preventing the occurrence and progression of fatty liver [28]. It can prevent the accumulation of triglycerides in the liver and liver fibrosis through a variety of mechanisms. In our survey, boys had the highest prevalence, especially among students aged 13 to 14. This may be because it is the time of menarche for most girls, during which the ovaries begin to produce estrogen. Second, according to our questionnaire, boys had higher BMI values than girls (Supplementary table 3). The proportion of students with obesity was relatively large, especially abdominal obesity. In terms of diet, boys preferred sweet foods, high-fat and high-calorie foods. These fried foods will increase the body fat content and increase the metabolic burden on the liver. To our surprise, we found that there was a significant difference on whether breast milk was exclusively consumed in four months after birth. We still do not yet know whether breast milk consumption is related to the high prevalence among boys. However, the earlier addition of complementary foods will also increase the burden on the digestive system because of imperfect digestive function.

Although there was no significant difference in the overall prevalence among junior school students, middle school students, and high school students, the prevalence of severe fatty liver increased significantly as the rise of the learning stage. This result indicates that senior high school students have a higher risk of severe fatty liver. This may be because most senior high school students are under high learning pressure, stay up late, and are sedentary. Furthermore, staying up late will reduce the body's immunity, and the liver's metabolic capacity will also decline. A sedentary lifestyle and less activity cause the accumulation of fat. Therefore, high school students should be more aware to the occurrence of fatty liver and actively address this issue in a timely manner.

Some studies have reported that NAFLD will appear in a family aggregation situation. When exploring the relationship between the prevalence of NAFLD and family living habits, we found that parents have a major influence on whether their children will have fatty liver. A high BMI among parents will increase the risk of fatty liver in children. Part of the reason may be that some obesity is inherited. Another component may be that parents' high BMI is related to living and eating habits, which will imperceptibly affect children. Second, the education level of parents will also have a certain impact on their children’s fatty liver disease. Generally, parents who have a high level of education may have a relatively deep understanding or some awareness of health and diseases; in addition, the prevention and control of children's fatty liver and other diseases may be relatively good. For children and adolescents, exercise must be strengthened first; physical exercise is the most effective method to prevent and treat NAFLD. Physical exercise can reduce the formation of free fatty acids, inhibit insulin resistance, reduce the risk factors for NAFLD and even reverse the condition [29]. Our study also showed that children with fatty liver appear to engage in less exercise, which is consistent with the results of Mansour-Ghanaei R's study on adults. However, the difference is that we did not find a relationship between exercise intensity and fatty liver disease [30]. It has been reported that aerobic exercise and resistance exercise can reduce steatosis of NAFLD; people with poor cardiopulmonary function are more suited to resistance exercise [31]. Moreover, we should pay attention to eating habits and ensure a healthy diet. It is crucial to ensure the proper types and amounts of fruits and vegetables. Vegetables and fruits contain a large number of vitamins, which have antioxidant properties and can reduce the risk of liver steatosis [32]. Children and adolescents must control the frequency of desserts and sweet beverages because these foods contain a large amount of fructose; fructose can induce insulin resistance and promote fat deposition in the liver [33]. We found that snacks and fried foods are frequently eaten by children with fatty liver in our research. These are high-calorie foods that are difficult to digest and absorb. They will eventually be converted into fat stored in various parts of the body, which will increase the burden on the liver. In addition to the points above, a regular breakfast has a major effect on the prevention of fatty liver. We have found that eating breakfast less frequently increases the risk of fatty liver in children and adolescents. Studies have found that skipping breakfast can easily cause overweight and obesity [34]. This may be the reason why these students are prone to fatty liver. Furthermore, chewing as slowly as possible and eating carefully during meals can prevent excessive dietary intake and fat accumulation. At present, the relationship between sleep and NAFLD has not been determined. Some studies suggest that a short sleep time increases the risk of fatty liver, but our study did not find any correlation between sleep time and NAFLD. Finally, in our research, information that is publicly shared in communities and other places is obviously effective in preventing fatty liver. In that case, more parents can be aware of the harm of obesity and hope to obtain more health knowledge. So we need to strengthen the popularization of science to prevent the occurence of NAFLD in school-aged children and adolescents. As our survey results show, health promotion is very important for children and adolescents to prevent fatty liver. Therefore, schools should pay attention to regular education on fatty liver knowledge. In addition, while educating children, schools should pay attention to educating children’s parents, because the influence of family on children’s fatty liver is very important.

## Conclusions

The prevalence of NAFLD among school-aged children and adolescents aged 7 to 18 in Shenyang is higher than the national average. Through questionnaires, our study confirmed that NAFLD was closely related to lifestyle habits and parental factors. A larger amount of data are needed to more thoroughly explore the prevalence of NAFLD and lifestyle in children and adolescents in Shenyang.

## Supplementary Information

**Additional file 1: Supplementary Table 1. **Questionnaire of children 

**Additional file 2: Supplementary Table 2. **Questionnaire of adolescents

**Additional file 3:** **Supplementary Table 3.** Comparision of lifestyle between boys andgirls

## Data Availability

The datasets generated and/or analysed during the current study are not publicly available because the public data has not obtained the consent of the participants’ guardian, but the data acquisition and analysis have obtained the consent of the participants’ guardian. But they are available from the corresponding author on reasonable request.

## Abbreviations

MAFLD: Metabolic-associated fatty liver disease
NAFLD: Nonalcoholic fatty liver disease
NASH: Nonalcoholic steatohepatitis
BMI: Body mass index
WHR: Waist-to-hip ratio
