# Supplementary material for: Epidemiology and lifestyle survey of non-alcoholic fatty liver disease in school-age children and adolescents in Shenyang, Liaoning
Source: BMC Pediatr. 2022 May 17;22:286. doi: 10.1186/s12887-022-03351-w (PMC9112471; doi:10.1186/s12887-022-03351-w)
Supplement: Supplementary file 1 — Additional file 1: Supplementary Table 1. Questionnaire of children [file 12887_2022_3351_MOESM1_ESM.docx]

| Table 2. Questionnaire of adolescents | | | | | |
| --- | --- | --- | --- | --- | --- |
|  |  |  | non-NAFLD (45) | NAFLD (8) | *P* value |
| Parental situation | BMI of father(kg/m^2^) |  | 22.47±2.88 | 25.18±3.86 | 0.032 |
|  | BMI of mother(kg/m^2^) |  | 21.25±2.22 | 24.54±3.67 | 0.002 |
|  | Education background (father) | High school and below | 18/45(40%) | 7/8(87.5%) | 0.013 |
|  |  | undergraduate and above | 27/45(60%) | 1/8(12.5%) |  |
|  | Education background (mather) | High school and below | 21/45(46.67%) | 6/8(75%) | 0.140 |
|  |  | undergraduate and above | 24/45(53.33%) | 2/8(25%) |  |
| The situation of birth (children) | Weight (kg) |  | 3.86±0.84 | 3.61±1.10 | 0.461 |
|  | Breast milk | Yes | 43/45(95.56%) | 7/7(100%) | 0.569 |
|  |  | No | 2/45(4.44%) | 0 |  |
| Movement of children | Sports | Like | 13/45(28.89%) | 5/8(62.5%) | 0.064 |
|  |  | Dislike | 32/45(71.11%) | 3/8(37.5%) |  |
|  | The frequnce of doing sports | Every day | 40/45(88.89%) | 6/8(75%) | 0.285 |
|  |  | Never | 5/45(11.11%) | 2/8(25%) |  |
|  | The time of sports every day | Less than 30min | 25/45(55.56%) | 7/8(75%) | 0.089 |
|  |  | More than 30min | 20/45(44.44%) | 1/8(25%) |  |
|  | Intensity of doing sports | Light exercise | 29/44(65.91%) | 5/8(62.5%) | 0.852 |
|  |  | Intense exercise | 15/44(34.09%) | 3/8(37.50%) |  |
|  | Sports grade | Good | 37/42(88.10%) | 3/8(37.5%) | 0.001 |
|  |  | Bad | 5/42(11.90%) | 5/8(62.5%) |  |
| Entertainment and rest of children | The time of sleeping every day(h) |  | 6.97±1.91 | 8.13±3.18 | 0.163 |
|  | the time of static activity every day(h) |  | 10.94±6.60 | 7.43±5.77 | 0.190 |
|  | The time of dynamic activity every day(h) |  | 2.05±1.56 | 3.17±1.84 | 0.116 |
| Diet of children | The frequence of eating fruit | More than once a day | 33/44(75%) | 6/8(75%) | 1.000 |
|  |  | Less than once a day | 11/44(25%) | 2/12(25%) |  |
|  | Kinds of fruits everyday if eating everyday |  | 2.27±1.17 | 2.25±1.04 | 0.959 |
|  | Kinds of fruits a week if not eating everyday? |  | 3.67±1.43 | 4.29±2.56 | 0.376 |
|  | The frequence of eating vegetable | More than once a day | 38/45(84.44%) | 8/8(100%) | 0.231 |
|  |  | Less than once a day | 7/45(15.56%) | 0 |  |
|  | Kinds of vegetable everyday if eating everyday |  | 2.67±1.71 | 2.25±0.71 | 0.503 |
|  | Kinds of vegetable a week if you not eating everyday |  | 4.47±2.70 | 3.29±0.76 | 0.261 |
|  | The frequnce of drinking sweet beverage a week | More than once a week | 34/45(75.56%) | 7/8(87.5%) | 0.457 |
|  |  | Less than once a week | 11/45(24.44%) | 1/8(12.50%) |  |
|  | Times of drinking sweet beverage every day if drinking every day |  | 1.00±0.92 | 0.57±0.54 | 0.244 |
|  | The frequnce of eating sweet food | More than once a week | 36/45(80%) | 8/8(100%) | 0.165 |
|  |  | Less than once a week | 9/45(20%) | 0 |  |
|  | The frequence of eating breakfast | Every day | 36/44(81.82%) | 4/8(50%) | 0.049 |
|  |  | 2-6 days a week | 8/44(18.18%) | 2/8(25%) | 0.653 |
|  |  | 1 day a week | 0 | 1/8(12.50%) | 0.018 |
|  |  | Not eating or long time | 0 | 1/8(12.50%) | 0.018 |
|  | The frequence of eating snacks | Every day | 6/45(13.33%) | 1/8(12.50%) | 0.949 |
|  |  | 1-6 days a week | 31/45(68.89%) | 7/8(87.5%) | 0.282 |
|  |  | Never | 8/45(17.78%) | 0 | 0.196 |
|  | The frequence of eating carefully and slowly | Always | 42/45(93.33%) | 7/8(87.5%) | 0.565 |
|  |  | Never | 3/45(6.66%) | 1/8(12.50%) |  |
|  | Picky eaters | No | 29/41(70.73%) | 3/7(42.86%) | 0.148 |
|  |  | Yes | 12/41(29.27%) | 4/7(57.14%) |  |
|  | The frequence of eating fast food | More than three times a week | 14/45(31.11%) | 2/8(25%) | 0.729 |
|  |  | Less than three times a week | 31/45(68.89%) | 6/8(75%) |  |
|  | The frequence of eating fried food | More than three times a week | 12/45(26.67%) | 2/8(25%) | 0.922 |
|  |  | Less than three times a week | 33/45(73.33%) | 6/8(75%) |  |
| Learning condition of children | The time in class(studying) |  | 10.80±3.13 | 9.86±3.34 | 0.467 |
|  | The time out of class(studying) |  | 2.93±1.76 | 2.71±1.70 | 0.766 |
|  | Study stress | Yes | 40/44(90.91%) | 7/7(100%) | 0.406 |
|  |  | No | 4/44(9.09%) | 0 |  |
| The situation of parental awareness | Do parents hope that children should be fat? | yes | 4/45(8.89%) | 2/8(25%) | 0.185 |
|  |  | no | 41/45(91.11%) | 6/8(75%) |  |
|  | Does community have information on nutrition and health? | Yes | 35/44(79.55%) | 3/8(37.5%) | 0.014 |
|  |  | No | 9/44(20.45%) | 5/8(62.5%) |  |
