# Supplementary material for: Epidemiology and lifestyle survey of non-alcoholic fatty liver disease in school-age children and adolescents in Shenyang, Liaoning
Source: BMC Pediatr. 2022 May 17;22:286. doi: 10.1186/s12887-022-03351-w (PMC9112471; doi:10.1186/s12887-022-03351-w)
Supplement: Supplementary file 2 — Additional file 2: Supplementary Table 2. Questionnaire of adolescents [file 12887_2022_3351_MOESM2_ESM.docx]

| Table 3. Comparision of lifestyle between boys and girls | | | | | |
| --- | --- | --- | --- | --- | --- |
|  |  |  | Boys | Girls | *P* value |
| Parental situation | BMI of father(kg/m^2^) |  | 23.84±2.95 | 23.33±3.97 | 0.452 |
|  | BMI of mother(kg/m^2^) |  | 22.82±3.49 | 22.10±3.25 | 0.265 |
|  | Education background (father) | High school and below | 25/62(40.32%) | 21/53(39.62%) | 0.939 |
|  |  | undergraduate and above | 37/62(59.68%) | 32/53(60.38%) |  |
|  | Education background (mather) | High school and below | 27/63(42.86%) | 17/53(32.08%) | 0.233 |
|  |  | undergraduate and above | 36/63(57.14%) | 36/53(67.92%) |  |
| The situation of birth (children) | Weight (kg) |  | 3.71±0.88 | 3.70±0.79 | 0.941 |
|  | Breast milk | Yes | 55/58(94.8%) | 48/51(94.1%) | 0.871 |
|  |  | No | 3/58(5.2%) | 3/51(5.9%) |  |
|  | whether breast milk was exclusively consumed in the first four months after birth | Yes | 33/58(56.9%) | 36/47(76.6%) | 0.034 |
|  |  | No | 25/58(43.1%) | 11/47(23.4%) |  |
| Movement of children | Sports | Like | 13/63(20.63%) | 27/54(50%) | 0.001 |
|  |  | Dislike | 50/63(79.37%) | 27/54(50%) |  |
|  | The frequnce of doing sports | Every day | 37/63(58.73%) | 15/54(27.78%) | 0.001 |
|  |  | 1-6 days a week | 18/63(28.57%) | 33/54(61.11%) | <0.001 |
|  |  | Never | 8/63(12.7%) | 6/54(11/1%) | 0.792 |
|  | The time of sports every day | Less than 30min | 28/63(44.44%) | 39/54(72.22%) | 0.002 |
|  |  | More than 30min | 35/63(55.56%) | 15/54(27.78%) |  |
|  | Intensity of doing sports | Light exercise | 19/62(30.6%) | 15/52(28.8%) | <0.001 |
|  |  | Moderate exercise | 11/62(17.7%) | 30/52(57.7%) |  |
|  |  | Intense exercise | 32/62(51.6%) | 7/52(13.5%) |  |
|  | Sports grade | Good | 43/55(78.18%) | 40/49(81.63%) | 0.662 |
|  |  | Bad | 12/55(21.82%) | 9/49(18.37%) |  |
| Entertainment and rest of children | The time of sleeping every day(h) |  | 7.87±2.07 | 6.87±1.73 | 0.006 |
|  | the time of static activity every day(h) |  | 9.59±6.17 | 10.16±6.00 | 0.638 |
|  | The time of dynamic activity every day(h) |  | 2.69±2.96 | 2.67±2.73 | 0.965 |
| Diet of children | The frequence of eating fruit | More than once a day | 52/63(82.54%) | 45/53(84.91%) | 0.732 |
|  |  | Less than once a day | 11/63(17.46%) | 8/53(15.09%) |  |
|  | Kinds of fruits everyday if eating everyday |  | 2.67±1.45 | 2.28±0.99 | 0.112 |
|  | Kinds of fruits a week if not eating everyday? |  | 3.91±1.91 | 4.24±2.17 | 0.470 |
|  | The frequence of eating vegetable | More than once a day | 56/63(88.89%) | 49/54(90.74%) | 0.742 |
|  |  | Less than once a day | 7/63(11.11%) | 5/54(9.26%) |  |
|  | Kinds of vegetable everyday if eating everyday |  | 3.05±1.79 | 2.88±1.67 | 0.609 |
|  | Kinds of vegetable a week if you not eating everyday |  | 4.42±2.21 | 4.66±2.75 | 0.667 |
|  | The frequnce of drinking sweet beverage a week | More than once a week | 49/63(77.78%) | 27/54(50%) | 0.002 |
|  |  | Less than once a week | 14/63(22.2%) | 27/54(50%) |  |
|  | Times of drinking sweet beverage every day if drinking every day |  | 0.73±0.87 | 0.73±0.72 | 0.992 |
|  | The frequnce of eating sweet food | More than once a week | 49/61(80.33%) | 40/54(74.07%) | 0.424 |
|  |  | Less than once a week | 12/61(19.7%) | 14/54(25.9%) |  |
|  | The frequence of eating breakfast | Every day | 55/63(87.3%) | 36/52(69.2%) | 0.018 |
|  |  | 2-6 days a week | 6/63(9.52%) | 14/52(26.92%) | 0.014 |
|  |  | 1 day a week | 1/63(1.6%) | 0/52 | 0.362 |
|  |  | Not eating or long time | 1/63(1.6%) | 2/52(3.8%) | 0.449 |
|  | The frequence of eating snacks | Every day | 8/62(12.9%) | 2/53(3.8%) | 0.083 |
|  |  | 1-6 days a week | 45/62(72.58%) | 45/53(84.91%) | 0.110 |
|  |  | Never | 9/62(14.5%) | 6/53(11.3%) | 0.612 |
|  | The frequence of eating carefully and slowly | Always | 56/63(88.9%) | 50/54(92.59%) | 0.494 |
|  |  | Never | 7/63(11.1%) | 4/54(7.4%) |  |
|  | Picky eaters | No | 46/57(80.7%) | 35/50(70.0%) | 0.198 |
|  |  | Yes | 11/57(19.3%) | 15/50(30.0%) |  |
|  | The frequence of eating fast food | More than three times a week | 5/63(7.94%) | 2/54(3.7%) | 0.336 |
|  |  | Less than three times a week | 58/63(92.06%) | 52/54(96.3%) |  |
|  | The frequence of eating fried food | More than three times a week | 21/63(33.33%) | 5/54(9.26%) | 0.002 |
|  |  | Less than three times a week | 42/63(66.67%) | 49/54(90.74%) |  |
| Learning condition of children | The time in class(studying) |  | 10.39±3.32 | 9.47±3.17 | 0.16 |
|  | The time out of class(studying) |  | 2.98±1.86 | 2.98±1.79 | 0.99 |
|  | Study stress | Yes | 54/61(88.52%) | 47/54(87.04%) | 0.808 |
|  |  | No | 7/61(11.5%) | 7/54(13.0%) |  |
