# Supplementary material for: Epidemiology and lifestyle survey of non-alcoholic fatty liver disease in school-age children and adolescents in Shenyang, Liaoning
Source: BMC Pediatr. 2022 May 17;22:286. doi: 10.1186/s12887-022-03351-w (PMC9112471; doi:10.1186/s12887-022-03351-w)
Supplement: Supplementary file 3 — Additional file 3: Supplementary Table 3. Comparision of lifestyle between boys andgirls [file 12887_2022_3351_MOESM3_ESM.docx]

| Table 1. Questionnaire of children | | | | | |
| --- | --- | --- | --- | --- | --- |
|  |  |  | non-NAFLD | NAFLD | *P* value |
| Parental situation | BMI of father(kg/m^2^) |  | 24.15±3.79 | 25.16±2.15 | 0.442 |
|  | BMI of mother(kg/m^2^) |  | 22.66±3.81 | 25.76±2.59 | 0.017 |
|  | Education background (father) | High school and below | 14/50(28%) | 7/12(58.33%) | 0.046 |
|  |  | undergraduate and above | 36/50(72%) | 5/12(41.67%) |  |
|  | Education background (mather) | High school and below | 24/51(47.06%) | 7/12(58.33%) | 0.482 |
|  |  | undergraduate and above | 27/51(52.94%) | 5/12(41.67%) |  |
| The situation of birth (children) | Weight(kg) |  | 3.50±0.77 | 4.08±0.78 | 0.056 |
|  | Breast milk | Yes | 42/46(91.30%) | 11/11(100%) | 0.310 |
|  |  | No | 4/46(8.70%) | 0 |  |
| Movement of children | Sports | Like | 18/52(34.62%) | 4/12(33.33%) | 0.933 |
|  |  | Dislike | 34/52(65.38%) | 8/12(66.67%) |  |
|  | The frequnce of doing sports | Every day | 49/52(94.23%) | 8/12(66.67%) | 0.006 |
|  |  | Never | 3/52(5.77%) | 4/12(33.33%) |  |
|  | The time of sports every day | Less than 30min | 31/52(59.62%) | 4/12(33.33%) | 0.099 |
|  |  | More than 30min | 21/52(40.38%) | 8/12(66.67%) |  |
|  | Intensity of doing sports | Light exercise | 34/50(68%) | 7/12(58.33%) | 0.525 |
|  |  | Intense exercise | 16/50(32%) | 5/12(41.67%) |  |
|  | Sports grade | Good | 44/44(100%) | 9/10(90%) | 0.034 |
|  |  | Bad | 0 | 1/10(10%) |  |
| Entertainment and rest of children | The time of sleeping every day(h) |  | 7.44±1.87 | 8.36±1.21 | 0.122 |
|  | the time of static activity every day(h) |  | 8.84±5.76 | 11.44±3.97 | 0.120 |
|  | The time of dynamic activity every day(h) |  | 3.23±3.72 | 2.81±3.16 | 0.770 |
| Diet of children | The frequence of eating fruit | More than once a day | 45/52(86.54%) | 10/12(83.33%) | 0.773 |
|  |  | Less than once a day | 7/52(13.46%) | 2/12(16.67%) |  |
|  | Kinds of fruits everyday if eating everyday |  | 2.49±1.12 | 3.55±1.92 | 0.105 |
|  | Kinds of fruits a week if not eating everyday? |  | 4.46±2.39 | 3.80±1.93 | 0.431 |
|  | The frequence of eating vegetable | More than once a day | 48/52(92.31%) | 11/12(91.67%) | 0.941 |
|  |  | Less than once a day | 4/52(7.69%) | 1/12(8.33%) |  |
|  | Kinds of vegetable everyday if eating everyday |  | 3.11±1.63 | 4.20±2.30 | 0.080 |
|  | Kinds of vegetable a week if you not eating everyday |  | 4.97±2.53 | 4.11±1.90 | 0.351 |
|  | The frequnce of drinking sweet beverage a week | More than once a week | 26/52(50%) | 9/12(75%) | 0.117 |
|  |  | Less than once a week | 26/52(50%) | 3/12(25%) |  |
|  | Times of drinking sweet beverage every day if drinking every day |  | 0.47±0.56 | 0.75±1.04 | 0.301 |
|  | The frequnce of eating sweet food | More than once a week | 38/50(76%) | 7/12(58.33%) | 0.218 |
|  |  | Less than once a week | 12/50(24%) | 5/12(41.67%) |  |
|  | The frequence of eating breakfast | Every day | 41/51(80.39%) | 10/12(83.33%) | 0.815 |
|  |  | 2-6 days a week | 8/51(15.69%) | 0/12 | 0.142 |
|  |  | 1 day a week | 0 | 1/12(8.33%) | 0.038 |
|  |  | Not eating or long time | 2/51(3.92%) | 1/12(8.33%) | 0.518 |
|  | The frequence of eating snacks | Every day | 1/50(2%) | 2/12(16.67%) | 0.033 |
|  |  | 1-6 days a week | 42/50(84%) | 10/12(83.33%) | 0.955 |
|  |  | Never | 7/50(14%) | 0 | 0.169 |
|  | The frequence of eating carefully and slowly | Always | 49/52(94.23%) | 8/12(66.67%) | 0.006 |
|  |  | Never | 3/52(5.77%) | 4/12(33.33%) |  |
|  | Picky eaters | No | 38/47(80.85%) | 11/12(91.67%) | 0.373 |
|  |  | Yes | 9/47(19.15%) | 1/12(8.33%) |  |
|  | The frequence of eating fast food | More than three times a week | 0 | 1/12(8.33%) | 0.036 |
|  |  | Less than three times a week | 52/52(100%) | 11/12(91.67%) |  |
|  | The frequence of eating fried food | More than three times a week | 7/52(13.46%) | 3/12(25%) | 0.321 |
|  |  | Less than three times a week | 45/52(86.54%) | 9/12(75%) |  |
| Learning condition of children | The time in class(studying) |  | 8.91±2.72 | 9.60±1.78 | 0.456 |
|  | The time out of class(studying) |  | 2.77±1.79 | 4.00±2.21 | 0.064 |
|  | Study stress | Yes | 44/52(84.62%) | 10/12(83.33%) | 0.912 |
|  |  | No | 8/52(15.38%) | 2/12(16.67%) |  |
| The situation of parental awareness | Do parents hope that children should be fat? | yes | 4/52(7.69%) | 1/12(8.33%) | 0.941 |
|  |  | no | 48/52(92.31%) | 11/12(91.67%) |  |
|  | Does community have information on nutrition and health? | Yes | 41/50(82%) | 8/11(72.73%) | 0.484 |
|  |  | No | 9/50(18%) | 3/11(27.27%) |  |
